# Controlled Fabrication of pH-Visualised Silk Fibroin–Sericin Dual-Network Hydrogels for Urine Detection in Diapers

**DOI:** 10.3390/gels11080671

**Published:** 2025-08-21

**Authors:** Yuxi Liu, Kejing Zhan, Jiacheng Chen, Yu Dong, Tao Yan, Xin Zhang, Zhijuan Pan

**Affiliations:** 1 College of Textile and Clothing Engineering, Soochow University, Suzhou 215021, Chinayantao@suda.edu.cn (T.Y.); 2 Key Laboratory of Jiangsu Province for Silk Engineering, Soochow University, Suzhou 215123, China; 3 National Engineering Laboratory for Modern Silk, Suzhou 215123, China

**Keywords:** fibroin–sericin, hydrogel, pH-visualised, urine detection, smart diaper

## Abstract

Urine pH serves as an indicator of systemic acid–base balance and helps detect early-stage urinary and renal disorders. However, conventional monitoring methods rely on instruments or manual procedures, limiting their use among vulnerable groups such as infants and bedridden elderly individuals. In this study, a pH-responsive smart hydrogel was developed and integrated into diapers to enable real-time, equipment-free, and visually interpretable urine pH monitoring. An optimised degumming process enabled one-step preparation of a silk fibroin–sericin aqueous solution. We employed a visible light-induced photo-crosslinking strategy to fabricate a dual-network hydrogel with enhanced strength and stability. Increasing sericin content accelerated gelation (≤15 min) and improved performance, achieving a maximum stress of 54 kPa, strain of 168%, and water absorption of 566%. We incorporated natural anthocyanins and fine-tuned them to produce four distinct colour changes in response to urine pH, with significantly improved colour differentiation (ΔE). Upon contact with urine, the hydrogel displays green within the normal pH range, indicating a healthy state. At the same time, a reddish-purple or blue colour serves as a visual warning of abnormal acidity or alkalinity. This intelligent hydrogel system combines rapid gelation, excellent mechanical properties, and a sensitive visual response, offering a promising platform for body fluid monitoring.

## 1. Introduction

Rising health awareness has driven demand for convenient and precise physiological health monitoring. Urine pH is a key indicator of health, reflecting changes in the body’s acid–base balance [1,2,3], as it directly correlates with renal excretion processes that regulate hydrogen ion and bicarbonate levels [4,5]. Acidic urine is typically a clinical correlation, often linked to high-protein diets, urinary tract infections, diabetes, and ketoacidosis, where increased acid load or organic acid excretion lowers urinary pH [6,7,8]; conversely, alkaline urine may occur in urea-splitting bacterial infections or kidney diseases that reduce acid excretion, and therefore, urine pH can act as a valuable early warning indicator for potential health conditions [9,10]. Compared to blood tests, urine pH tests offer the advantages of easy sampling and non-invasiveness, making them suitable for routine monitoring [11,12,13]. However, current urine analysis methods face limitations in sensitivity, convenience, and real-time performance. They often rely on specialised equipment or manual operation, restricting their applicability in daily health monitoring. Specific vulnerable populations—such as bedridden elderly people, infants, and children with special needs—often cannot express discomfort. As a result, latent health issues like urinary tract infections may go unnoticed, leading to delayed intervention and disease progression. In such cases, visualising hidden health conditions becomes particularly important.

Diapers, as a daily wearable product, offer multiple advantages for urine monitoring, including direct contact with urine, broad usage scenarios, and ease of integrating sensing functions such as real-time monitoring of urine pH, glucose, infection markers, and other biomarkers. These features make them an ideal platform for wearable biosensor systems and integrated flexible electronics in health monitoring [14,15,16]. However, conventional diapers are limited to basic functions such as urine absorption and simple wetness indication. These features are insufficient to meet the increasing demand for daily health monitoring. In this context, integrating real-time urine pH monitoring into diapers enables early detection of conditions such as urinary tract infections. It also helps prevent skin complications caused by prolonged wear without timely replacement. The design of such smart diapers improves the efficiency and convenience of urine monitoring, offering more precise health management for vulnerable populations. This approach holds significant social value and broad application prospects.

Hydrogels, due to their unique three-dimensional network structure, can effectively absorb and retain water while maintaining softness and elasticity [17]. They can withstand external mechanical stress such as stretching and compression [18,19,20]. These properties render hydrogels widely utilised in various fields, including drug sustained release, tissue engineering, water purification, and intelligent sensing. In the biomedical field in particular, their excellent compatibility with human tissues has made them a foundational material for many innovative applications [21]. Silk fibroin, a natural polymer, exhibits outstanding biocompatibility and favourable mechanical strength, making it an ideal material in biomedical applications. Its abundant functional groups, such as hydroxyl and carboxyl groups, confer high structural tunability [22,23] to silk fibroin hydrogels, enabling their integration with various functional molecules to meet diverse application needs [24]. Leveraging this advantage, silk fibroin hydrogels are capable of effectively loading pH-responsive molecules, allowing them to respond to changes in urine pH sensitively and thus demonstrating strong potential for non-invasive urine pH monitoring in wearable healthcare systems.

Silk fibroin hydrogels are primarily prepared using physical [25], chemical, and photo-crosslinking methods [26]. Physical methods, such as temperature variation or solvent exchange, can induce the self-assembly of silk fibroin into hydrogels. However, these approaches offer limited control over key structural parameters such as pore size, crosslink density, and network homogeneity, which are critical for determining hydrogel mechanical performance and diffusion characteristics [27]. Chemical methods achieve crosslinking through chemical crosslinkers to enhance the mechanical properties of hydrogels [28,29,30]. However, they may compromise biocompatibility and biodegradability due to residual toxic crosslinkers and the formation of non-degradable chemical bonds. In contrast, photoinitiated crosslinking offers significant advantages in controlling the degree and spatial distribution of crosslinking. It enables precise tuning of hydrogel structure; avoids the side effects of chemical crosslinkers, such as cytotoxicity, potential inflammatory responses, and long-term tissue irritation; and features rapid reaction kinetics with simple operation [31]. Typical photoinitiators include horseradish peroxidase (HRP) [32,33], riboflavin phosphate sodium (FMN) [34], PEGDMA [35], PEGDA [36], poly(N-vinylcaprolactam), curcumin [37], diaryliodonium hexafluorophosphate (DPI), glutaraldehyde (GTA) [38,39], tyrosinase, genipin [40,41], etc. Among commonly used photoinitiators, riboflavin (vitamin B2) stands out as a natural photosensitiser [34,42] with excellent UV responsiveness and biocompatibility [43]. It is widely employed in the photo-crosslinking of silk fibroin hydrogels. Riboflavin enables crosslinking under mild conditions [44], enhances the stability and mechanical strength of the hydrogel, and offers good biodegradability, making it well-suited for biomedical applications [45].

The network structure of hydrogels plays a decisive role in determining their performance. Single-network silk fibroin-based hydrogels exhibit relatively simple internal structures. During gelation, silk fibroin molecules tend to shift from a random coiled conformation to a β-sheet structure, forming dense and rigid β-sheet aggregates [46]. This structural characteristic results in critical drawbacks, including insufficient mechanical strength and significant brittleness. These issues severely limit the practicality of the material in applications that require both strong mechanical properties and flexibility [47]. Introducing additional polymer materials to create a double-network structure can significantly improve mechanical properties and application viability. However, the inclusion of these foreign materials often presents challenges, such as decreased biocompatibility or increased complexity in degradation products.

In contrast, sericin, a natural polymer homologous to silk fibroin, inherently possesses excellent biocompatibility. Its molecular conformation primarily consists of random coils, with an open chain structure that is highly flexible, providing the material with outstanding plasticity. Sericin is rich in long polar amino acid side chains (e.g., serine, aspartic acid) and hydrophilic groups (-OH, -COOH, -NH_2_, etc.), and it thus exhibits excellent moisture-regulating and moisturising properties [48]. Introducing sericin into the silk fibroin hydrogel system not only significantly improves brittleness but also synergistically enhances the toughness and overall elasticity of the hydrogel [49]. This synergistic effect, originating from homologous materials, notably improves the composite hydrogel’s overall mechanical properties without introducing potentially incompatible components. As a result, it demonstrates distinct advantages in application fields with stringent requirements for material flexibility, mechanical strength, and biocompatibility, such as biomedical implants, flexible sensors, and wearable devices.

Current preparation methods for sericin-silk fibroin composite materials rely on complex, multi-step extraction processes. Traditional approaches typically involve the separate extraction of silk fibroin and sericin, followed by mixing [50]. These methods require multiple steps, such as dissolution and purification. They are cumbersome, costly, and demand strict process control, including temperature, pH, and timing. During purification, exogenous impurities or reagents, such as salts, organic solvents, and potential contaminants, may be introduced, which can compromise the biocompatibility and safety of the proteins. Another method involves purifying and drying the aqueous sericin extract obtained after the degumming process to produce soluble sericin powder. This yields high purity but is time-consuming. It also depends on harsh conditions, like high temperatures or strong alkalis, which can cause uncontrolled degradation of sericin, leading to reduced molecular weight, broader distribution, and disruption of molecular structures, such as complex globular conformations. As a result, the functional activity and application value of sericin are significantly diminishing. Overall, the lack of an efficient, mild one-step co-extraction strategy results in low process efficiency for traditional methods.

In summary, this study has developed a novel stimuli-responsive hydrogel material utilising silk fibroin (SF) and sericin (Seri). The core innovation lies in precisely controlling the parameters of degumming time and the concentration of sodium carbonate (the degumming agent) in the silk degumming process, which enables tuneable sericin retention and efficient preparation of SF-Seri mixed solutions. We selected biocompatible natural riboflavin (RB) as the photo-crosslinking agent to construct photo-crosslinked SF-Seri/RB hydrogel networks. Key preparation parameters were systematically optimised, including sericin content, precursor solution pH, and irradiation time. These optimisations effectively tuned the hydrogel’s pore structure, mechanical properties, and liquid absorption capacity. To enable urine monitoring, we incorporated natural anthocyanin (Cy), extracted from red cabbage, as a pH-responsive molecule. Optimisation of the Cy loading process further improved the pH-responsive behaviour of SF-Seri/RB@Cy in urine environments. Hydrogel-integrated diapers offer urine absorption and visualisation, along with pH monitoring through colourimetric responses, enabling timely alerts when urinary pH exceeds the normal range. The novelty of this work stems from fully leveraging the inherent biocompatibility, tunability, and photo-crosslinking properties of the SF-Seri system. We propose a new strategy for preparing high-performance smart hydrogels. This strategy and the developed materials hold significant potential in health monitoring applications, particularly for continuous or on-demand urine analysis and personal health management devices.

## 2. Results and Discussion

### 2.1. Construction of Double-Network SF-Seri/RB Hydrogels

#### 2.1.1. Fabrication of a Single-Network SF/RB Hydrogel

To investigate the effect of RB concentration on the gelation behaviour of silk fibroin (SF) solution, the SF concentration will be fixed at 10 wt.%. Figure S1 indicates that when the RB concentration was below 1 mg/mL, no gel formed. When the RB concentration reached 1 mg/mL, we measured the gelation time to be 40 min. As the RB concentration increased to 1.5 mg/mL, the gelation time remarkably shortened to 25 min. However, when we elevated RB concentration to 2.0 mg/mL, the gelation time extended to 30 min. Utilising the gelation rate as the key evaluation criterion, the optimal RB concentration is 1.5 mg/mL. Under this optimal RB concentration condition, we carried out further investigations to explore the effect of SF concentration on the gelation process. When the SF concentration increased from 10 wt.% to 15 wt.%, it indicated that higher SF content promotes gelation. Nevertheless, when the SF concentration rose to 20 wt.%, the gelation time prolonged to 20 min, suggesting that an overly high SF concentration inhibited the gelation rate.

As a photosensitive crosslinking agent, the concentration of RB exerts a significant influence on the intermolecular crosslinking efficiency. Within the range of 1.5 to 2.0 mg/mL, the intermolecular spacing of RB is optimal for forming an effective crosslinked network. Hydrogen bonding and hydrophobic interactions between SF chains and RB molecules act synergistically to promote the gelation process. At low RB concentrations, insufficient crosslinking points limit gelation efficiency. Conversely, when the RB concentration is too high, molecular crowding occurs, increasing steric hindrance, which restricts molecular diffusion and rearrangement, ultimately reducing crosslinking efficiency. Similarly, the impact of SF concentration on the gelation process also exhibits a biphasic characteristic. Moderately increasing the SF concentration (5~15 wt.%) reduces the molecular chain spacing and lowers the energy barrier required for crosslinking, facilitating the formation of a stable network. However, when the SF concentration exceeds 15 wt.%, the high concentration restricts the movement of molecular chains and reduces the spatial matching efficiency, consequently slowing down the gelation rate.

In summary, the cooperative crosslinking behaviour between RB and SF molecules is significantly affected by concentration regulation. An appropriate molecular spacing and chain segment flexibility are crucial for efficiently constructing a gel network. The optimised gelation conditions for the single-network SF/RB hydrogel are 15 wt.% SF, 1.5 mg/mL RB, and 15 min of light exposure.

#### 2.1.2. Construction and Structure–Property Characterisation of Dual-Network SF-Seri/RB Hydrogels

**Figure 1:**
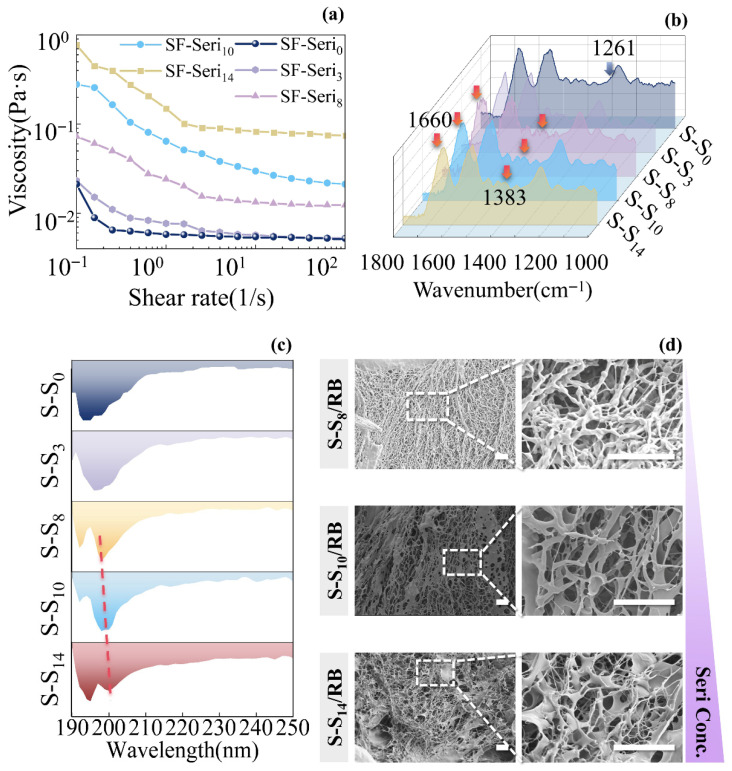
Fabrication of photo-crosslinked SF-Seri/RB hydrogels. (**a**) Shear rate–viscosity curves of SF-Seri solutions; (**b**) FTIR spectra of SF-Seri solutions; (**c**) CD spectra of SF-Seri solutions(The dotted line represents the offset of the negative peak); (**d**) SEM images of the network structure in SF-Seri/RB hydrogels.

We obtained SF-Seri aqueous solutions by directly dissolving SF fibres containing varying residual amounts of sericin. The shear viscosity–shear rate curves of the resulting solutions are illustrated in Figure 1a. Shear viscosity, a physical parameter quantifying a fluid’s resistance to flow, is higher for greater viscosity. As the Sericin content increased, the shear viscosity of the hydrogel significantly rose. Compared to the zero-shear viscosity of the pure SF solution (SF-Seri_0_, 0.02 Pa·s), the zero-shear viscosity of the SF-Seri_14_ solution reached 0.77 Pa·s, representing a 38-fold increase. Fourier transform infrared spectroscopy (FTIR) analysis of SF-Seri solutions is presented in Figure 1b. Characteristic absorption peaks of sericin at 1383 cm^−1^. This peak was absent in SF-Seri_0_, barely detectable in SF-Seri_3_, but visible in SF-Seri_14_.

Furthermore, the characteristic peak at 1261 cm^−1^ (amide III) in SF-Seri_14_ notably diminished, while the peak at 1660 cm^−1^ (amide I) markedly intensified, which indicates a decrease in β-sheet structure and a rise in amorphous structure. Circular dichroism (CD) spectra of SF-Seri solutions (Figure 1c) revealed a single negative peak in SF-Seri_0_ and SF-Seri_3_, typical of β-sheets. In contrast, SF-Seri_8_, SF-Seri_10_, and SF-Seri_14_ displayed double negative peaks, suggesting an α-helix structure [51]. As the sericin content increased, the negative peaks gradually shifted to longer wavelengths, indicating a partial transition [52] from β-sheet to α-helix structures [53]. Among them, SF-Seri_14_ exhibited the most vigorous negative peak intensity and the most pronounced α-helix characteristic peak. The increase in α-helix content facilitates hydrogel formation by improving mechanical strength, structural stability, and hydration capacity [54]. To evaluate the effect of sericin content on the gelation performance of SF-Seri/RB hydrogels, three formulations—SF-Seri_8_/RB, SF-Seri_10_/RB, and SF-Seri_14_/RB—were prepared. These hydrogels exhibit excellent moldability and can be shaped into arbitrary forms. Their representative appearances are shown in Figure S2a. At a low sericin content (SF-Seri_8_/RB), the hydrogel displayed fine, fibrous pore walls with relatively small and uniformly distributed pores. At a medium sericin content (SF-Seri_10_/RB), the pore walls transitioned into lamellar structures, featuring increased pore size and a relatively loose structure. At a high content (SF-Seri_14_/RB), a complex network structure emerged, with both fibrous and lamellar pore walls coexisting, creating a more developed three-dimensional porous network (Figure 1d). As the content of sericin increased, the proportion of α-helix and amorphous structures gradually rose, resulting in looser and more random molecular chain arrangements, which enhanced the flexibility and plasticity of the hydrogel network. Concurrently, the increased sericin content provided more cross-linking sites, promoting greater cross-linking density, altering network morphology, and improving hydrogel stability. Rheological data can also prove this point. In the absence of sericin (Seri), the viscosity of the SF/RB hydrogel precursor solution is relatively low, and its tan(δ) (Figure S2c) curve shows a gradual increase, indicating that a single-network structure predominantly controls the hydrogel.

Additionally, the mechanical strength of the SF/RB hydrogel is extremely weak, and it cannot be effectively tested, which further suggests that its network structure is fragile and lacks the reinforcing effect of a second network. As the content of sericin (Seri) increases, the viscosity of the precursor solution significantly increases, indicating that the introduction of the second network enhances the system’s viscosity. After preparation into hydrogel form, the tan(δ) curve of the SF-Seri_3_/RB dual-network hydrogel begins to exhibit a plateau in the low-frequency region, indicating the emergence of the second network’s effect. As the content of sericin increases, the tan(δ) curve of the SF-Seri/RB dual-network hydrogel gradually becomes flatter. Mechanical data indicated that a higher sericin content yielded superior mechanical performance (see Figure S2d). Based on these results, we selected SF-Seri_10_/RB and SF-Seri_14_/RB for subsequent experiments.

**Figure 2:**
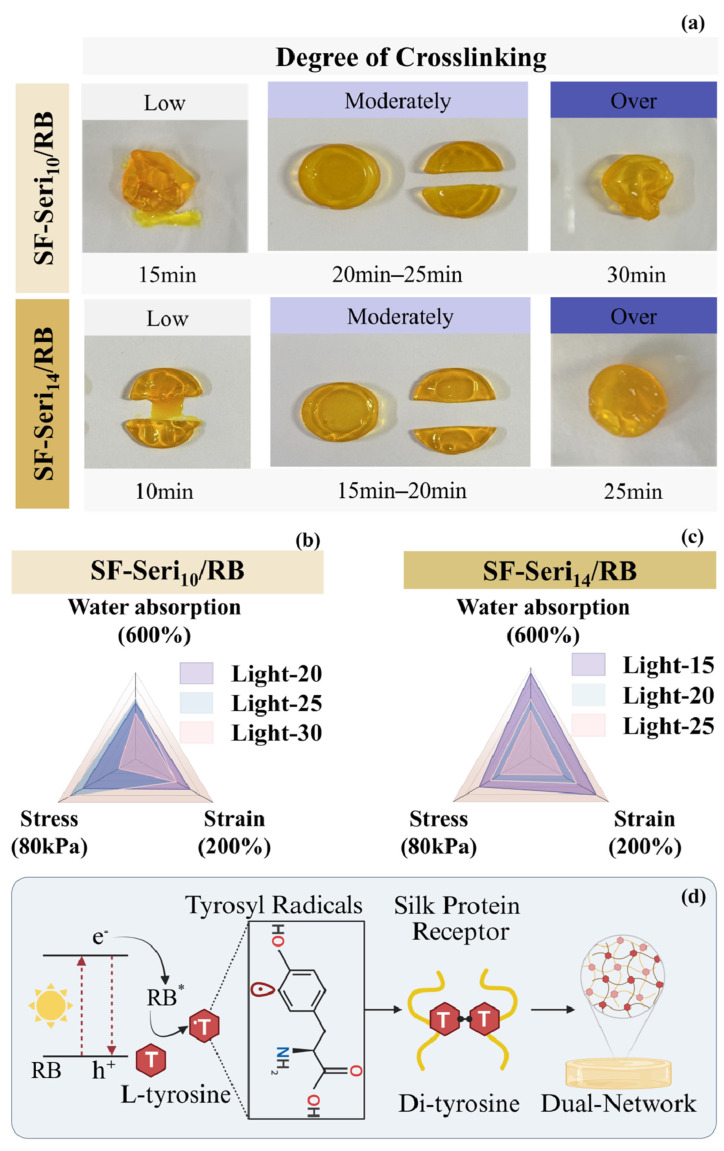
Structural–mechanical modulation of photo-crosslinked SF-Seri/RB hydrogels. (**a**) Influence of illumination time on the moulding efficacy of SF-Seri/RB hydrogels. Effects of illumination time on the mechanical and water absorption properties of (**b**) SF-Seri_10_/RB hydrogels and (**c**) SF-Seri_14_/RB hydrogels. (**d**) Schematic of the photo-crosslinking mechanism: L-tyrosine radicals form dityrosine bonds in silk protein receptors, facilitating the formation of SF-Seri dual-network hydrogels. (S-S represents SF-Seri).

Due to the limited penetration depth of light [55], hydrogels exposed to short irradiation times undergo crosslinking primarily at the surface, while the internal regions remain uncrosslinked or only partially crosslinked. As the irradiation time increases, the hydrogel gradually attains a moderately crosslinked state, forming an internal network structure with adequate strength and flexibility [56]. This structure offers favourable plasticity and elasticity, enabling the hydrogel to maintain well-defined cutting edges during sectioning. However, excessive crosslinking caused by prolonged light exposure results in an overly compact and flattened hydrogel network structure, as shown in Figure 2a, which leads to surface shrinkage and uneven deformation, significantly compromising flatness. The internal chemical composition and the distribution density of photosensitive groups influence the crosslinking rate. Consequently, hydrogels with varying sericin contents necessitate different illumination times to attain the same degree of crosslinking. SF-Seri_10_/RB hydrogel requires 20~25 min of visible light exposure for complete gelation, whereas SF-Seri_14_/RB hydrogel only requires 15~20 min. Crosslinking degree significantly affects the internal structure and mechanical properties of hydrogels [56]. With prolonged irradiation, the water absorption and strength of SF-Seri_10_/RB hydrogel initially increase and then decrease. At the same time, the breaking strain continues to decline, reaching a minimum of nearly 100% (Figure 2b). The SF-Seri_14_/RB hydrogel achieves complete crosslinking within 15 min of irradiation, with maximum values of stress (~54 kPa), strain (~168%), and swelling ratio (~566%) (Figure 2c). However, with further increase in illumination time, these parameters significantly decrease (to approximately 32 kPa, 80%, and 312%, respectively). Considering the mechanical properties and crosslinking efficiency of the hydrogel, we selected the optimal sample for subsequent experiments as the SF-Seri_14_/RB hydrogel with an illumination time of 15 min.

The photo-crosslinking mechanism of SF-Seri/RB hydrogel is initially driven by the excitation of the photosensitiser RB under visible light (Figure 2d). Upon irradiation, RB transitions to an excited state (RB*), which reacts with the hydroxyl groups of tyrosine residues in the silk protein receptor (SPR), generating tyrosyl radicals (T) [57]. The tyrosyl radicals subsequently form dityrosine bonds (T-T) [58], driving the crosslinking of silk fibroin chains [43]. Based on this, the crosslinking between sericin and SF forms a second network that plays a key role [59]. Both proteins contain tyrosine residues capable of crosslinking [52], enabling further interactions and a more complex network. Collectively, the synergistic effects of RB-initiated photo-crosslinking and sericin–SF molecular interactions result in a multilayered network architecture, which significantly improves the overall performance and stability of the hydrogel.

### 2.2. Optimisation of SF-Seri/RB Hydrogel Fabrication

#### 2.2.1. Influence of Precursor Solution Parameters on Gel Network Formation and Mechanical Properties

Environmental temperature significantly influences the microstructure [60] of SF-Seri_14_/RB hydrogels. As illustrated in Figure S3a, at 10 °C, the hydrogel network appears sparse and loosely arranged in a fibrous formation. Upon increasing the temperature to 15 °C, a honeycomb-like pore wall structure begins to form; however, most pore walls remain closed with poor connectivity, resulting in a discontinuous overall network. At 20 °C, the pore size and distribution become more uniform. Still, we observe partial collapse of the pore walls, and the three-dimensional architecture lacks sufficient definition, resulting in a collapse of the structure and poor spatial organisation. Upon reaching 25 °C, the hydrogel develops a well-defined three-dimensional porous structure with rough pore walls, a higher number of pores, and significantly enhanced interconnectivity. Further increasing the temperature to 30 °C, the lamellar structure becomes overly compact, the interconnectivity between pores decreases, the uniformity of pore size distribution worsens, and some regions display smooth surfaces lacking visible porosity.

Regarding the mechanical properties (as depicted in Figure S3b), as the temperature rises, both breaking strain and breaking stress initially decrease from 10 °C to 15 °C and then increase, with the breaking strain decreasing again at 30 °C. Considering both pore morphology and mechanical performance, the hydrogel crosslinked at 25 °C demonstrates optimal fracture strain and stress, achieving a balance between strength and flexibility. Therefore, we selected 25 °C as the optimal crosslinking temperature for subsequent experiments.

In acidic environments (Figure S3c), reducing the pH from 6.3 causes the SF-Seri/RB hydrogel to shift gradually from a 3D network to a planar structure. The pore size gradually decreases, and the morphology shifted from regular circular shapes to irregular shapes. We reduced the pH to 5, the hydrogel structure contracted and collapsed, resulting in a more compact and denser pore architecture. Mechanical tests (Figure S3d) reveal that as the solution pH decreases, both the stress and strain of the hydrogel increase initially and then decrease. Notably, at a pH of 5.5, the hydrogel achieves optimal tensile strength and elasticity (97 kPa, 153%). Consequently, we selected samples prepared at a pH of 5.5 for further investigation.

Under alkaline conditions (Figure S3e), adjusting the precursor pH also significantly impacts hydrogel formation. At pH 7, the hydrogel displays large, unevenly distributed internal pores, with pore walls showing clear signs of stretching and tearing, as seen in the SEM image from Figure S3e, indicating ductile tearing and confirming tensile rupture. When we increased the pH to 7.5, the pore distribution became more uniform, though certain irregularities remain. At pH 8, a well-organised honeycomb-like porous structure emerges, characterised by large pore size, high connectivity, and smooth, intact pore walls. Upon further increasing the pH to 9, the pore walls become denser, the pore size decreases substantially, and structural collapse and adhesion result in poor interconnectivity. Mechanical testing (Figure S3f) indicates that as the pH increases, the stress of the hydrogel rises, reflecting an increase in stiffness. At the same time, the strain continues to decrease, indicating a reduction in flexibility. Considering both the porous structure and mechanical performance, the hydrogel prepared at a pH of 8 exhibits the most regular pore morphology and is the representative sample for studying gelation mechanisms under alkaline conditions.

#### 2.2.2. Effect of pH on Pore Morphology and Network Properties of SF-Seri/RB Hydrogels

To investigate the influence of precursor solution pH on the gelation behaviour of SF-Seri/RB hydrogels, samples with pH values of 5.5 and 8.0 were selected to represent weakly acidic and weakly alkaline conditions, respectively. In contrast, the original sample with a pH of 6.5 was the standard control. These three samples were designated as follows: weakly acidic hydrogel (SF-Seri/RB-A, pH 5.5), standard hydrogel (SF-Seri/RB, pH 6.5), and weakly basic hydrogel (SF-Seri/RB-B, pH 8.0). By comparing and analysing these three representative samples, we systematically evaluated the effects of precursor solution pH on the hydrogel’s structure and properties.

**Figure 3:**
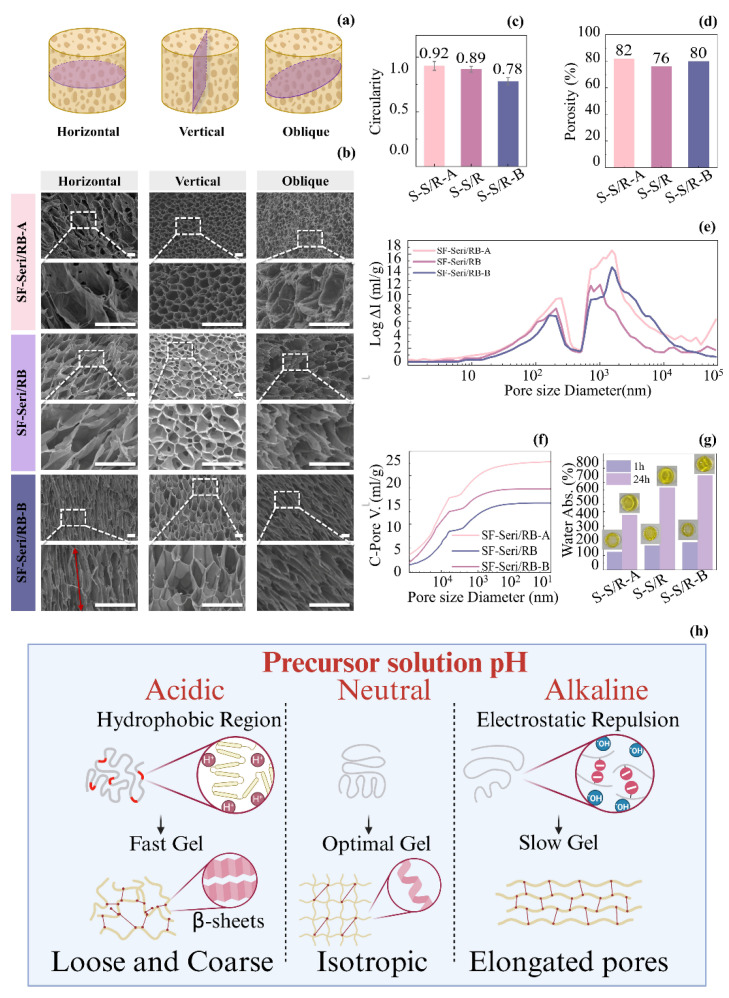
Regulation of the SF-Seri/RB hydrogel network structure by the pH of the precursor solution. (**a**) Schematic illustration of cross-sectional orientations for SF-Seri/RB hydrogel analysis. (**b**) SEM images of the SF-Seri/RB hydrogel microstructure from different sectioning orientations (The red arrow represents the direction of pore distribution morphology). (**c**) Analysis of pore circularity index. (**d**) Comparison of porosity. (**e**) Pore size distribution curves. (**f**) Cumulative pore volume curves. (**g**) Comparison of swelling ratios at 1 h and 24 h. (**h**) Schematic illustration of the structural regulation mechanism of the SF-Seri/RB hydrogel mediated by the pH of the precursor solution.

Three hydrogel samples underwent freeze-drying treatment, and their cross-sectional morphologies in three directions—horizontal, vertical, and oblique—were captured (Figure 3a). Through multi-directional observation (Figure 3b), we systematically analysed the pore structural characteristics of the various hydrogels. The pore morphology of SF-Seri/RB-A was closest to a circular shape, with a circularity index of 0.92 (Figure 3c); however, significant structural differences among the three cross-sectional orientations. In the horizontal section, we observed large pores with soft walls and a dense pore structure. In the vertical section, the pore size was notably smaller, and the structure became denser. The oblique section showed collapsed pore structures with closed pores, indicating pronounced anisotropy. The standard SF-Seri/RB sample displayed a honeycomb-like pore structure, characterised by a circularity index of 0.89. The structural differences between the horizontal and oblique sections were minimal, suggesting a relatively uniform pore architecture. The vertical cross-section revealed well-aligned pores with high interconnectivity, suggesting a relatively isotropic network structure. SF-Seri/RB-B exhibited a typical three-dimensional honeycomb structure with a circularity index of 0.78. The pore walls were well-defined and uniformly distributed. Elongated and highly interconnected pores were observed on both the transverse and oblique sections, indicating excellent structural integrity.

To obtain more accurate pore structure parameters, we performed mercury intrusion porosimetry (MIP) on the three SF-Seri/RB hydrogel formulations, with detailed data shown in Figure 3d–f. The results revealed that SF-Seri/RB-A exhibited the highest porosity, reaching 82%. It also demonstrated a broad pore size distribution (approximately 1000~10,000 nm) and the most significant cumulative pore volume, approaching 25 mL/g, indicating both large pore size and high pore volume. In contrast, the SF-Seri/RB sample showed the lowest porosity (76%) and the smallest pore volume (~15 mL/g), owing to its more uniform internal structure and narrower pore size distribution. Furthermore, the water absorption capacity of the hydrogels increased with the pH of the precursor solution. After 1 h, SF-Seri/RB-A showed the lowest water uptake (123.21%), while SF-Seri/RB-B achieved the highest (188.85%). After 24 h, the swelling ratio of SF-Seri/RB-B increased 3~4 fold, reaching 648.03%, which was nearly twice that of SF-Seri/RB-A (376.53%), indicating enhanced hydration behaviour.

The underlying mechanism by which the pH of the hydrogel precursor solution influences its structure and properties is illustrated in Figure 3h. In summary, an acidic environment promotes the exposure of hydrophobic regions within silk protein molecules, thereby enhancing intermolecular hydrogen bonding and hydrophobic interactions. These interactions facilitate the formation of stable β-sheet structures, which in turn increase hydrogel stress while reducing strain. When the environmental pH approaches the isoelectric point of silk fibroin, the enhanced intermolecular attractions accelerate aggregation and gelation. However, the resulting gel structures often exhibit inhomogeneous morphologies, characterised by larger pores and irregular pore walls, which significantly impair the hydrogel’s water absorption capacity. Conversely, in an alkaline environment, silk fibroin molecular chains acquire negative charges, leading to electrostatic repulsion that hinders rapid molecular association. This repulsive barrier delays gelation, allowing more time for the molecules to undergo ordered self-assembly. As a result, it forms a more uniform three-dimensional network with finer crosslinking points, featuring elongated honeycomb-like pore structures. This detailed structure not only enhances the mechanical stability of the gel but also improves its ability to absorb water by increasing the pore surface area and connectivity. Therefore, hydrogels in alkaline environments exhibit higher water absorption, reflected by higher swelling and absorption rates. In neutral conditions (pH = 6.5), the positive and negative charges on the silk protein molecules are roughly balanced, and the system is near its isoelectric state. However, due to the ionic strength and adjustments in the solution environment, the aggregation process is slightly slower than in the acidic environment, allowing the protein chains more time to rearrange and locally order. This moderate gelation rate helps form a more regular three-dimensional network structure with uniformly distributed pores, good isotropy, and connectivity. The gel structure in this neutral environment strikes a balance between aggregation rate and structural order, maintaining a good equilibrium between mechanical strength and water absorption capacity.

### 2.3. Construction and Evaluation of pH-Responsive SF-Seri/RB@Cy Hydrogels

#### 2.3.1. Analysis of Anthocyanin Loading and Colourimetric Response Performance

**Figure 4:**
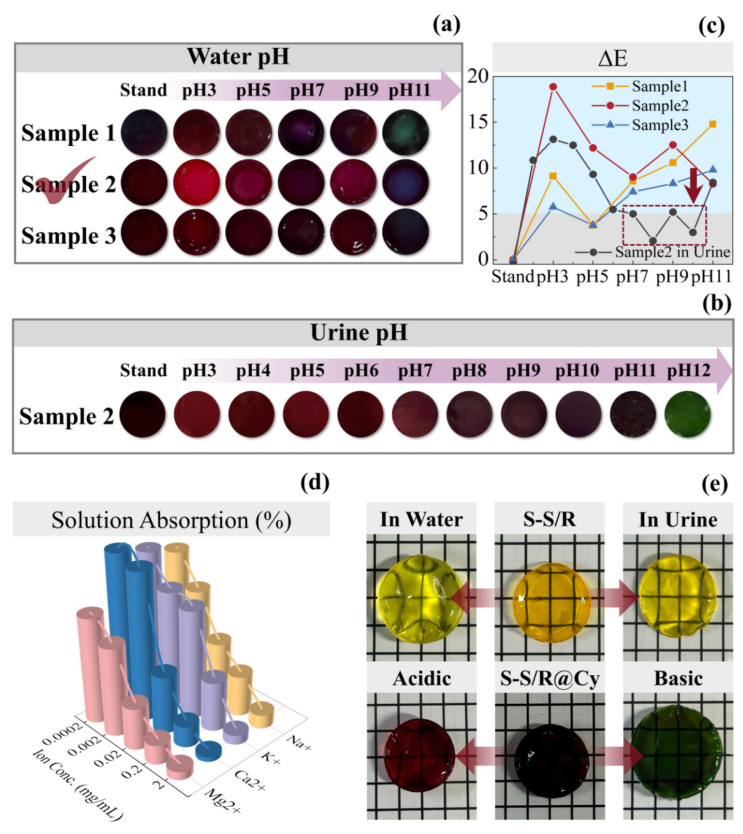
Colourimetric response behaviour of SF-Seri/RB@Cy hydrogels in different liquid environments. (**a**) Colour response of hydrogel samples in aqueous solutions with varying pH values (The optimal sample has been annotated in the figure). (**b**) Colour response of hydrogel samples in artificial urine at different pH levels. (**c**) ΔE curves indicating colour difference. (**d**) Comparison of liquid absorption rates in ionic solutions with varying ion concentrations. (**e**) Swelling comparison of SF-Seri/RB hydrogels in water and artificial urine and of SF-Seri/RB@Cy hydrogels in acidic and basic artificial urine.

The SF-Seri/RB-A, SF-Seri/RB, and SF-Seri/RB-B hydrogels were immersed in Cy solution to obtain SF-Seri/RB@Cy hydrogels, shown in Figure S4a, designated as Sample 1, Sample 2, and Sample 3, respectively. Upon anthocyanin loading, the hydrogels transitioned to a deep purple colour and exhibited a certain degree of swelling. Figure S4b illustrates the anthocyanin loading rates of Samples 1 to 3. The results indicate that Sample 3 achieved a loading rate of 140.01%, demonstrating that the SF-Seri/RB hydrogel prepared under weakly alkaline conditions exhibits superior loading capacity, which aligns with the adsorption properties of hydrogel materials in solution. The mechanical properties of the hydrogels slightly decreased after anthocyanin incorporation, with specific data provided in Figure S4c,d. Further analysis of the colour-changing behaviour of SF-Seri/RB@Cy hydrogels under different pH conditions is shown in Figure 4a. As the pH increased, Sample 1 gradually changed from dark red to green. Sample 2 transitioned from bright red to dark purple, and eventually to blue. In contrast, Sample 3 exhibited minimal colour change, primarily appearing dark red and black. Consequently, we selected Sample 2 for subsequent experiments.

Sample 2 was immersed in urine to observe its pH-responsive colour change (Figure 4b). The experiment found that when the pH of the urine was acidic, the SF-Seri/RB@Cy hydrogel appeared red. As the pH increased to 10, the sample’s colour transitioned from red to dark red. With a further pH increase to 11–12, the colour gradually shifted from dark green to green, with minimal overall colour change. To quantify the colour variation, we employed the ΔE (Delta E) value as the colour difference metric, defined by the International Commission on Illumination (CIE). A smaller ΔE value indicates a less noticeable colour difference, while a larger ΔE value signifies a more significant colour change. Figure 4c shows the variation in ΔE values of SF-Seri/RB@Cy hydrogels after immersion in solutions with different pH values. At pH 5, both Sample 1 and Sample 3 exhibited ΔE values below 5, indicating minimal colour change under mildly acidic conditions, which suggests a limited ability to achieve an effective colourimetric response across the whole pH range.

In contrast, Sample 2 displayed superior colourimetric performance, with ΔE values consistently above 5 in all aqueous environments. Its responsiveness was significantly higher than that of the other two samples. However, when we immersed Sample 2 in urine, the ΔE values dropped below 5 within the pH range of 7 to 11, which indicates limited colour change under physiological conditions, which may be difficult to detect by the naked eye. Therefore, further tuning of the hydrogel’s pH responsiveness is necessary to enhance visual detectability.

To elucidate the origin of the differential pH-responsive colour changes of hydrogels in water and artificial urine, we investigated the influence of metal ions (Na^+^, K^+^, Mg^2+^, and Ca^2+^) commonly present in urine. We immersed SF-Seri/RB hydrogels in aqueous solutions containing varying concentrations of these ions. The results showed a concentration-dependent decrease in water uptake, with divalent cations—particularly Ca^2+^ and Mg^2+^—exerting a more pronounced inhibitory effect (Figure 4d). This phenomenon is likely attributed to ionic cross-linking between the functional groups of silk proteins and metal ions, especially divalent species, which enhances the compactness of the hydrogel network. The denser structure restricts water infiltration from urine, thereby reducing the hydrogel’s swelling and attenuating its visible colour response. To verify this hypothesis, we further compared the swelling behaviours of SF-Seri/RB hydrogels in water and artificial urine (Figure 4e). The hydrogels exhibited significantly lower water uptake and weaker expansion in urine, supporting the role of ion-induced network densification. Additionally, we observed a pH-dependent volume change in urine: the hydrogels shrank in acidic conditions and expanded under basic conditions. Based on these findings, we propose two strategies to improve the pH responsiveness of SF-Seri/RB@Cy hydrogels in urine. First, adjusting the pH of the anthocyanin loading solution may facilitate the formation of a more expanded network, reducing excessive cross-linking upon contact with urinary ions and thereby preserving structural flexibility. Second, to avoid over-saturation of colour and improve visual resolution, we should moderately reduce the concentration of the Cy solution during loading. This adjustment ensures optimal colour visibility while maintaining hydrogel integrity and responsiveness.

#### 2.3.2. Regulation of Anthocyanin Loading Conditions on the Colourimetric Response of Hydrogels

**Figure 5:**
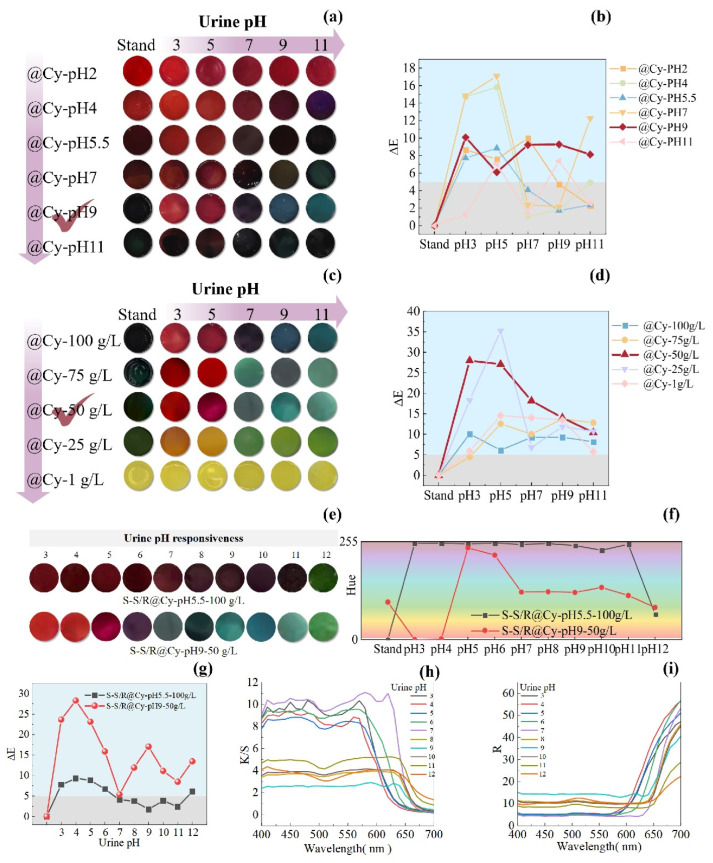
Regulation of colourimetric performance of SF-Seri/RB@Cy hydrogels by pH and concentration of anthocyanin solution. (**a**) Visual colour response of hydrogels loaded with anthocyanin solutions of varying pH. (**b**) Corresponding ΔE values as a function of pH. (**c**) Visual colour response of hydrogels loaded with anthocyanin solutions of different concentrations. (**d**) Corresponding ΔE curves. (**e**) Comparative colourimetric response of hydrogels under optimised and unoptimised loading conditions in urine. (**f**) Hue angle variations under different urine pH conditions. (**g**) Comparative ΔE values for optimised versus unoptimised samples. (**h**) K/S curve for optimised versus unoptimised samples. (**i**) R curve for optimised versus unoptimised samples.

We fixed the anthocyanin concentration at 100 g/L and gradually adjusted the pH of the anthocyanin solution. We labelled the hydrogel samples as @Cy-pH2, @Cy-pH4, @Cy-pH5.5, @Cy-pH7, @Cy-pH9, and @Cy-pH11. We immersed the sample in urine with different pH values, and the colour-changing effects are shown in Figure 5a. From @Cy-pH2 to @Cy-pH4, the SF-Seri/RB@Cy hydrogels predominantly displayed a red colour in urine across different pH ranges, which was indistinguishable to the naked eye. We observed from @Cy-pH5.5 to @Cy-pH7, a shift from red to darker hues. Notably, @Cy-pH9 exhibited a transformation from deep black to bright red (acidic urine), dark purple (in neutral urine), and blue-green shades (in alkaline urine). The @Cy-pH11 sample appeared black and did not display any colour-changing properties. The ΔE values of the SF-Seri/RB@Cy hydrogel samples (Figure 5b) indicate that only @Cy-pH9 had ΔE values greater than 5 across different urine pH conditions, signifying that the colour change was visible to the naked eye. Furthermore, @Cy-pH9’s colour change encompassed three colour systems: red, purple, and blue-green. Consequently, we chose @Cy-pH9as the optimal parameter.

We set the pH of the fixed anthocyanin solution to 9. We gradually reduced the concentration of the anthocyanin solution to enhance the vibrancy and chromatic richness of the colour change response. We presented the hydrogel sample codes as @Cy-100 g/L, @Cy-75 g/L, @Cy-50 g/L, @Cy-25 g/L, and @Cy-1 g/L. As the concentration decreased, the colour change response became increasingly diverse. Notably, the colour changes in @Cy-25 g/L, @Cy-50 g/L, and @Cy-75 g/L were particularly pronounced (Figure 5c). The original @Cy-50 g/L sample exhibited a deep green colour, transitioning to a bright red in acidic urine, a dark green in neutral urine, and a blue-green hue in alkaline urine, which aligns more closely with practical application requirements. When the anthocyanin solution concentration was less than 50 mg, the samples primarily displayed a yellow-green colour and lacked colour-changing functionality. The ΔE values of the SF-Seri/RB@Cy hydrogel samples are illustrated in Figure 5d. ΔE represents the total colour difference between two colours, indicating the degree of colour change, and the figure demonstrates that adjusting the concentration of Cy significantly improved the colour change effect of the hydrogel, with most samples exhibiting ΔE values greater than 5, indicating a visible colour change. The @Cy-50 g/L sample showed the most significant ΔE difference, making it the optimal parameter.

Based on these results, we determined the optimal conditions for Cy loading were a pH of 9 and a concentration of 50 g/L. The hydrogel prepared under these conditions (SF-Seri/RB@-CypH9-50 g/L) was compared with the unoptimised reference group (SF-Seri/RB@Cy-pH5.5-100 g/L) to evaluate pH-responsive behaviour in artificial urine. The reference hydrogel showed only a slight colour change from dark red to dark green, resulting in an overall dark appearance that was difficult to distinguish visually. In contrast, the optimised hydrogel exhibited a vivid and distinct colour transition: from bright red to purplish-red and eventually to bluish-green. The improved visual response confirms the effectiveness of the concentration and pH adjustments. The hue angle (H), a key parameter for quantifying colour tone in the CIE LAB colour space, was used to characterise the chromatic shifts further. The corresponding hue ranges for different colour categories are listed in Table S1. As shown in Figure 5f, the @Cy50 g/L hydrogel spanned four major hue categories during the pH response process. Moreover, its ΔE values were significantly higher than those of the unoptimised sample (Figure 5g), confirming the enhanced colourimetric sensitivity of the optimised system. The K/S curve generally decreases with increasing wavelength (Figure 5h), showing significant absorption in the blue-purple region (400–500 nm) and weaker absorption in the red region (600–700 nm), reflecting changes in spectral absorption intensity. The R curve shows a transition from low reflection (strong absorption) at the blue end to high reflection at the red end, with a shift in the reflection peak (Figure 5i). In the acidic urine environment (pH = 3–4), the K/S peak reaches 8–10, with the peak located between 400–550 nm, and R in the red region increases to 45–55, with the object displaying a red colour. In the weakly acidic urine environment (pH = 5–6), the K/S peak decreases to 8–9, with the peak shifting toward 550 nm, and R in the middle spectrum increases to about 50, with the object displaying a purple colour. In the neutral urine environment (pH = 7), the K/S value increases to 10–11, and R decreases to below 45, with the object showing a blue colour. In the weakly alkaline urine environment (pH = 8–9), the K/S value decreases to 2–4, the peak shifts to 550–650 nm, and R slightly decreases, with the object showing a blue-green colour. In the alkaline urine environment (pH = 10–12), the K/S value is 3–5, and R significantly decreases to about 20, with the object showing a green colour. Overall, as the pH increases, the K/S value gradually decreases, the reflection peak shifts toward longer wavelengths, and the colour transitions from the red spectrum to the green spectrum. Table 1 shows the advantages of the loading properties and pH response properties of the hydrogel substrates prepared in this study compared with other studies.

#### 2.3.3. Integration of Hydrogel in Diapers and Evaluation of Urine Detection Performance

**Figure 6:**
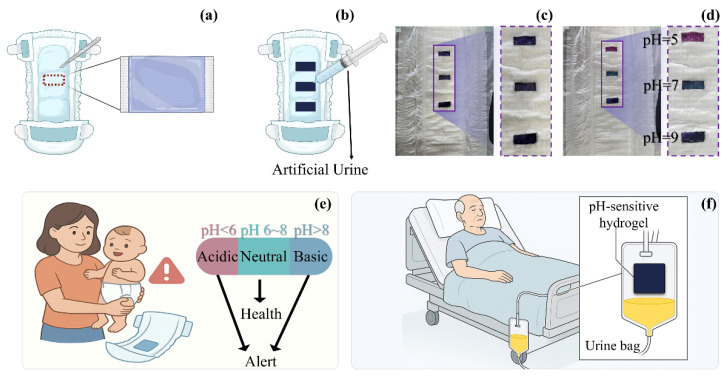
Design and application prospects of a pH-responsive hydrogel-integrated urine monitoring system for personalised care in special populations. (**a**) Schematic illustration of the integration method of pH-responsive hydrogel into diapers. (**b**) Diagram of the evaluation method for hydrogel-integrated diapers under pH-responsive conditions. (**c**) Illustration of hydrogel strip embedded within the diaper structure. (**d**) Photographic demonstration of pH-responsive effects at pH 5, 7, and 9. (**e**) Conceptual diagram showing health assessment and alert functionality for infant urine monitoring based on smart hydrogel. (**f**) Application prospect of smart hydrogel in geriatric urinary health monitoring.

To evaluate the feasibility of integration and the colourimetric responsiveness of SF-Seri/RB@Cy hydrogels in urine monitoring applications, we conducted a functional integration experiment using a disposable diaper as the substrate. Specifically, we partially removed the nonwoven surface layer of the diaper and evenly applied a pre-prepared hydrogel precursor solution to the absorbent core. Upon light irradiation, we formed a stable hydrogel film, followed by immersion in an anthocyanin solution to enable functional loading. The nonwoven layer was then repositioned and fixed in place, resulting in a thin, flexible SF-Seri/RB@Cy hydrogel coating on the diaper surface with both softness and sensing capabilities (Figure 6a). To assess the hydrogel’s pH-responsive behaviour further, we integrated three hydrogel films onto a single diaper and separately injected with artificial urine at pH values of 5, 7, and 9 (Figure 6b,c). The procedure is documented in Supplementary Video S1. Upon contact with the urine, the hydrogels exhibited rapid and visually distinguishable colour changes: acidic urine (pH 5) induced a purplish-red colour, neutral urine (pH 7) yielded green, and alkaline urine (pH 9) resulted in a blue hue, as shown in Figure 6d. These distinct colour transitions provide an intuitive and effective method for visual pH indication in real time. In practical healthcare applications, this hydrogel system shows broad potential for non-invasive physiological monitoring. For infants under three years old who still wear diapers, the appearance of a purplish-red signal may alert caregivers to reduce formula intake or animal protein consumption and to monitor for possible hyperglycemia, dehydration, enzyme deficiencies, or early signs of fever and diarrhoea (Figure 6e). Blue colouration may suggest alkaline urine, potentially associated with bacterial contamination and a risk of urinary tract infection. This platform is also applicable to elderly individuals with long-term immobility (Figure 6f). When integrated into nursing pads or catheter systems, the hydrogel can serve as a pH-responsive sensor, offering early alerts related to impaired renal function or increased susceptibility to urinary infections. Such integration enhances the intelligence and responsiveness of long-term care strategies.

## 3. Conclusions

In this study, we developed a novel pH-responsive intelligent hydrogel based on silk fibroin (SF) and sericin (Seri). We successfully integrated it into diapers to construct a wearable, equipment-free platform for real-time urine pH monitoring. By precisely controlling the silk degumming process, we achieved tuneable sericin retention and efficient preparation of SF-Seri composite solutions. Using natural riboflavin as a biocompatible photoinitiator, a double-network hydrogel was fabricated via visible light-induced photo-crosslinking, exhibiting excellent mechanical strength, rapid gelation (≤15 min), and high water absorption (566%). To enable colourimetric sensing of urine pH, we incorporated anthocyanins as pH-responsive indicators. The hydrogel displayed a clear, four-range colour transition with enhanced ΔE values, enabling intuitive visual recognition of physiological versus abnormal urine conditions. Specifically, green indicated healthy neutral urine, while reddish-purple and blue provided immediate visual alerts under acidic or alkaline conditions, respectively. The proposed strategy fully leverages the natural biocompatibility, tunability, and photoreactivity of the SF-Seri system. It offers an innovative approach for engineering high-performance smart hydrogels tailored for real-time, non-invasive body fluid monitoring. This work not only demonstrates the feasibility of integrating sensing and absorption into a single wearable platform but also holds significant promise for personalised health management applications, particularly among populations requiring continuous, at-home care.

## 4. Materials and Methods

### 4.1. Controlled Preparation of Degummed Silk Fibroin Fibres with Varied Sericin Contents

In the degumming process, anhydrous Na_2_CO_3_ (supplied by Shanghai ShaoYuan Reagent Co., Ltd, China.) was added to boiling deionised water at 100 °C, followed by the introduction of natural silk at a liquor-to-material ratio of 1:100. The concentrations of the Na_2_CO_3_ solution were set at 0.2 wt.% and 0.5 wt.%. In contrast, the degumming durations were varied at 15 min, 30 min, 45 min, and 60 min under boiling conditions. During the reaction, the solution was gently stirred with a glass rod every 15 min. Upon completion of the degumming treatment, the silk fibres were removed, rinsed thoroughly three times with deionised water to eliminate residual sericin, and excess moisture was carefully squeezed out. Subsequently, the fibres were spread evenly on clean aluminium foil and dried in a fume hood at room temperature for 24 h, ensuring that the quality stabilised and no further changes occurred, thus obtaining silk fibres with varying levels of sericin content. The mass of the silk fibres before and after degumming was recorded, and the residual sericin content was calculated based on the weight difference. The degumming parameters and corresponding sample codes are summarised in Table 2.

### 4.2. Construction of SF-Seri/RB Composite Hydrogel

The forming process parameters of the SF-Seri/RB hydrogel were determined by adjusting key factors such as riboflavin concentration, silk fibroin solution concentration, and sericin content, using gelation time as the evaluation criterion. To precisely regulate the pH of the SF-Seri/RB aqueous solution, sodium hydroxide solution (pH 10) and citric acid solution (pH 4) were mixed in varying proportions. The resulting solution was then poured into a silicone mould and exposed to a full-spectrum supplemental light source (COB integrated optical source, wavelength range 400–800 nm, 55 W) for photopolymerisation. The effects of irradiation duration and the initial pH of the precursor solution on the crosslinking degree, microstructure, and physicochemical properties of the hydrogel were systematically investigated.

### 4.3. Fabrication of pH-Responsive SF-Seri/RB@Cy Hydrogels

**Figure 7:**
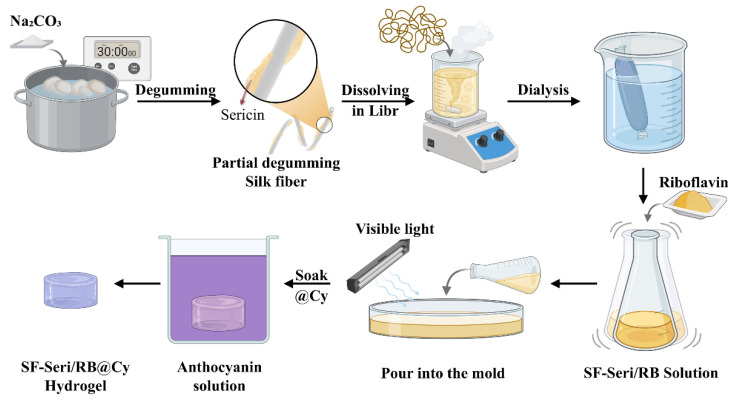
Schematic illustration of the preparation process of SF-Seri/RB@Cy hydrogel.

Dissolve anthocyanin in deionised water with stirring to ensure complete dissolution. Prepare anthocyanin solutions by adjusting the concentration and pH using sodium hydroxide (pH 10) and citric acid (pH 2). Immerse the SF-Seri/RB hydrogels in the anthocyanin solution. Store the colour-changing hydrogels at 4 °C for subsequent analysis. The specific preparation process is shown in Figure 7.

### 4.4. Physicochemical Characterisation and Functional Evaluation of the Hydrogels

The morphology of the hydrogels was observed using a Hitachi S-4800 scanning electron microscope (SEM) after freeze-drying, sectioning, and gold sputtering. Pore shape irregularity was quantified by circularity using ImageJ (https://imagej.net/ij/) (accessed on 30 July), where binarised images were segmented via the Watershed algorithm and analysed with the Measure function. Pore size distribution and porosity were further evaluated using a V9620 mercury intrusion porosimeter at 10 °C with a pressure range of 0.10–61,000.00 psia, analysing pores in the diameter range of 5 nm–100 μm using the Washburn equation for calculation. Rheological properties were assessed using a DHR-2 rotational rheometer equipped with a 20 mm parallel plate (gap: 1 mm), performing frequency sweep tests from 0.1 to 100 s^−1^. Mechanical properties were tested with an INSTRON-3365 universal testing machine at a stretching rate of 10 mm/min and a gauge length of 20 mm. Stress and strain were calculated from load-displacement data. FTIR spectra were collected using a Nicolet 5700 spectrometer (3500–300 cm^−1^, 32 scans, 4 cm^−1^ resolution). Circular dichroism (CD) spectra were recorded on a J-815 instrument (250–190 nm, 100 nm/min, N_2_ flow: 5 mL/min). Swelling behaviour was measured by immersing dried samples in deionised water for 24 h, recording mass changes until equilibrium, and the swelling ratio was expressed as mass-based. Anthocyanin loading was performed by soaking samples in a 100 mg/mL anthocyanin solution (pH 5.5) under the same procedure. Colourimetric response was evaluated using a colourimeter, with L*, a*, and b* values recorded in triplicate using a pulsed xenon lamp, colour-filtered to approximate a D65 light source and an observer angle of 90°.

## Figures and Tables

**Table 1:** Comparison of properties of different anthocyanin-loaded hydrogel substrates.

Hydrogel Components	Water Absorption	Anthocyanin Loading Method/Rate	Colour-Change Interval	Reference
PEGDA/LCNF	12.5%	soak/-	pH 7~14	[61]
Alginate	250%	mix/-	pH 4, 7, 9	[62]
SNF/SA	-	encapsulate/87.43%	pH 2~7, ΔE < 8	[63]
PVA/SA	-	Dye/-	pH 2~12	[64]
PUH-DCT	-	Mix/-	pH 5~9	[65]
Chitosan/Poly (vinyl alcohol)	Within 20 min, 40~120%	Mix/-	pH 1~12	[66]
Fmoc-FDFD	-	encapsulate/98.27 ± 0.87%	pH 1.5~6.8	[67]
SA/PCA/Fe	within an hour <25 g/g	encapsulate/62~82%	pH 2~9	[68]
SF-Seri/RB *	**Maximum 566%**	**Maximum 140** **%**	**pH 3~12,The minimum ΔE > 5**	**-**

* The hydrogel substrate prepared for this thesis.

**Table 2:** Code names of silk fibre samples with varying sericin content levels.

Sample Code	Na_2_CO_3_ Concentration (wt.%)	De-Gumming Time (min)	Debonding Rate (%)	Seri Content (%)
SF-Seri_28_	0.2	0	0	28
SF-Seri_14_		15	14	14
SF-Seri_10_		30	18	10
SF-Seri_8_		45	20	8
SF-Seri_3_		60	25	3
SF-Seri_0_	0.5	60	28	0

## Data Availability

The data presented in this study are openly available in the article.

## Supplementary Materials

The following supporting information can be downloaded at: https://www.mdpi.com/article/10.3390/gels11080671/s1, Figure S1: Regulation of SF/RB single-network hydrogel formation.; Figure S2: Characterisation of SF-Seri/RB hydrogels: moldability, mechanical properties, and microstructures.; Figure S3: Regulation of SF-Seri/RB hydrogel microstructure and mechanical properties by environmental temperature and precursor pH; Figure S4: Anthocyanin loading performance and its effect on the mechanical properties of hydrogels; Video S1: Feasibility of integration and the colourimetric responsiveness of SF-Seri/RB@Cy hydrogels in urine monitoring applications.
