# Supplementary material for: Controlled Fabrication of pH-Visualised Silk Fibroin–Sericin Dual-Network Hydrogels for Urine Detection in Diapers
Source: Gels. 2025 Aug 21;11(8):671. doi: 10.3390/gels11080671 (PMC12385573; doi:10.3390/gels11080671)
Supplement: Supplementary file 1 [file gels-11-00671-s001.zip › gels-3822716-supplementary.pdf]

Controlled Fabrication of pH-Visualised Silk Fibroin–Sericin Dual-Network Hydrogels for Urine Detection in Diapers

Yuxi Liu[1], Kejing Zhan[1], Jiacheng Chen[1], Yu Dong[1], Tao Yan[1,2,3], Xin Zhang[1,2,3], Zhijuan Pan[1,2,3*]*

[1]College of Textile and Clothing Engineering, Soochow University, Suzhou, Jiangsu 215021, China

[2]Key Laboratory of Jiangsu Province for Silk Engineering, Soochow University, Suzhou 215123, China

[3]National Engineering Laboratory for Modern Silk, Suzhou 215123, China

[*]Correspondence: zhangxin@suda.edu.cn(X.Z.), zhjpan@suda.edu.cn.(Z.P.)

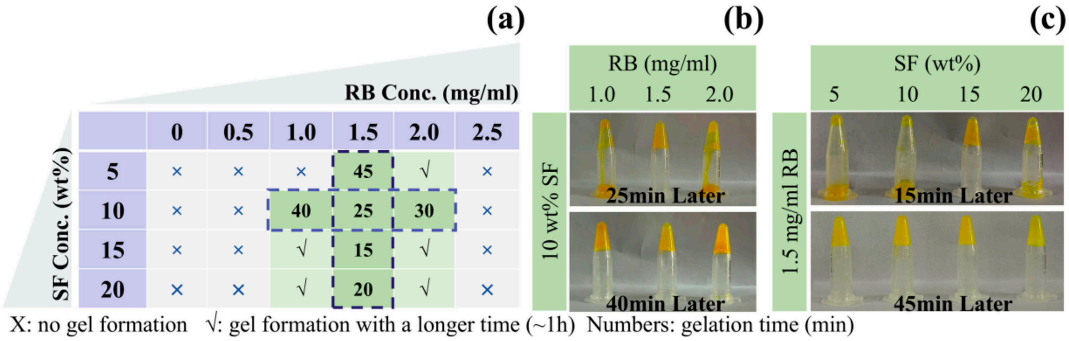

Figure S1. Regulation of SF/RB single-network hydrogel formation.

(a) Effect of SF and RB concentrations on gelation;

(b) Gelation outcomes at 10 wt% SF;

(c) Gelation outcomes at 1.5 mg/mL RB.

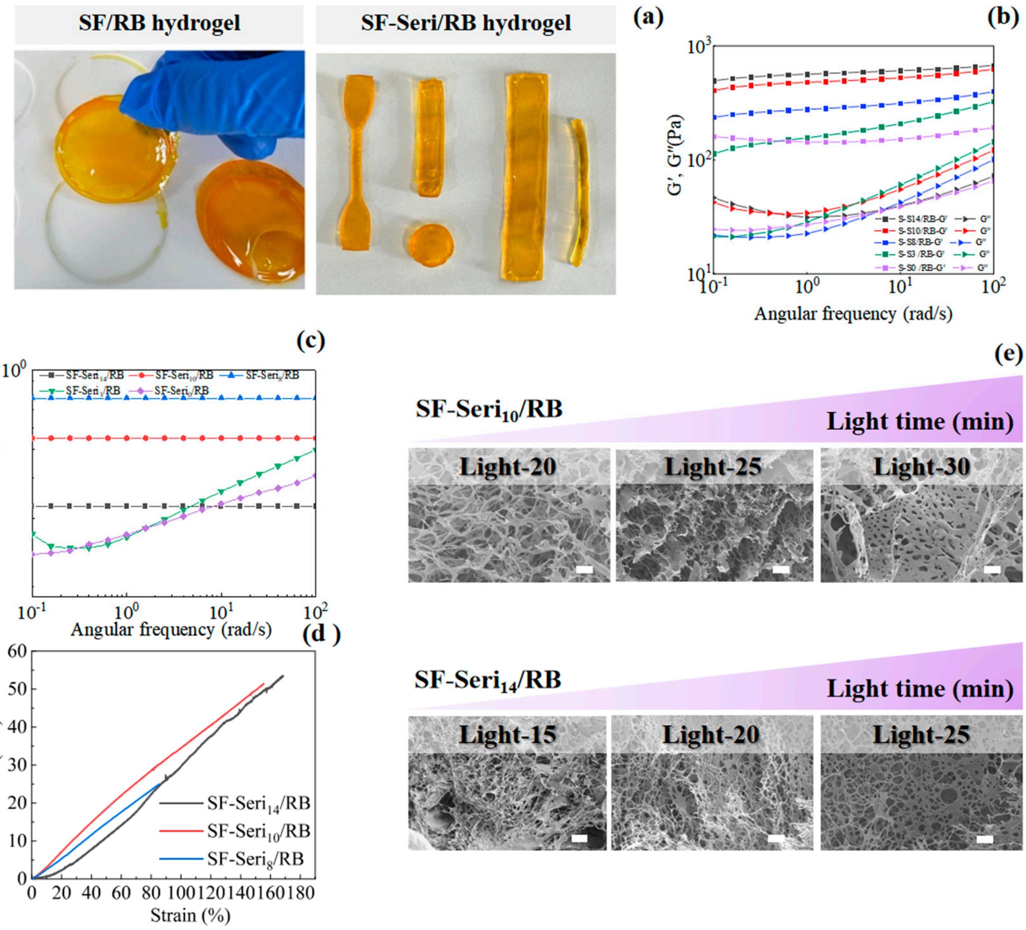

Figure S2. Characterization of SF-Seri/RB hydrogels: moldability, rheological property, mechanical properties, and microstructures.

(a) Photographs demonstrating Comparison pictures of shapes before and after adding seri and the moldability of SF-Seri/RB hydrogels into various shapes;

(b) G 'and G "curves of SF-Ser/RB hydrogels with different sericin contents.

(c) tan δ of SF-Ser/RB hydrogels with different sericin contents.

(d) Effect of sericin content on the mechanical strength of the hydrogels;

(e) SEM images showing the influence of light exposure time on hydrogel network architecture.

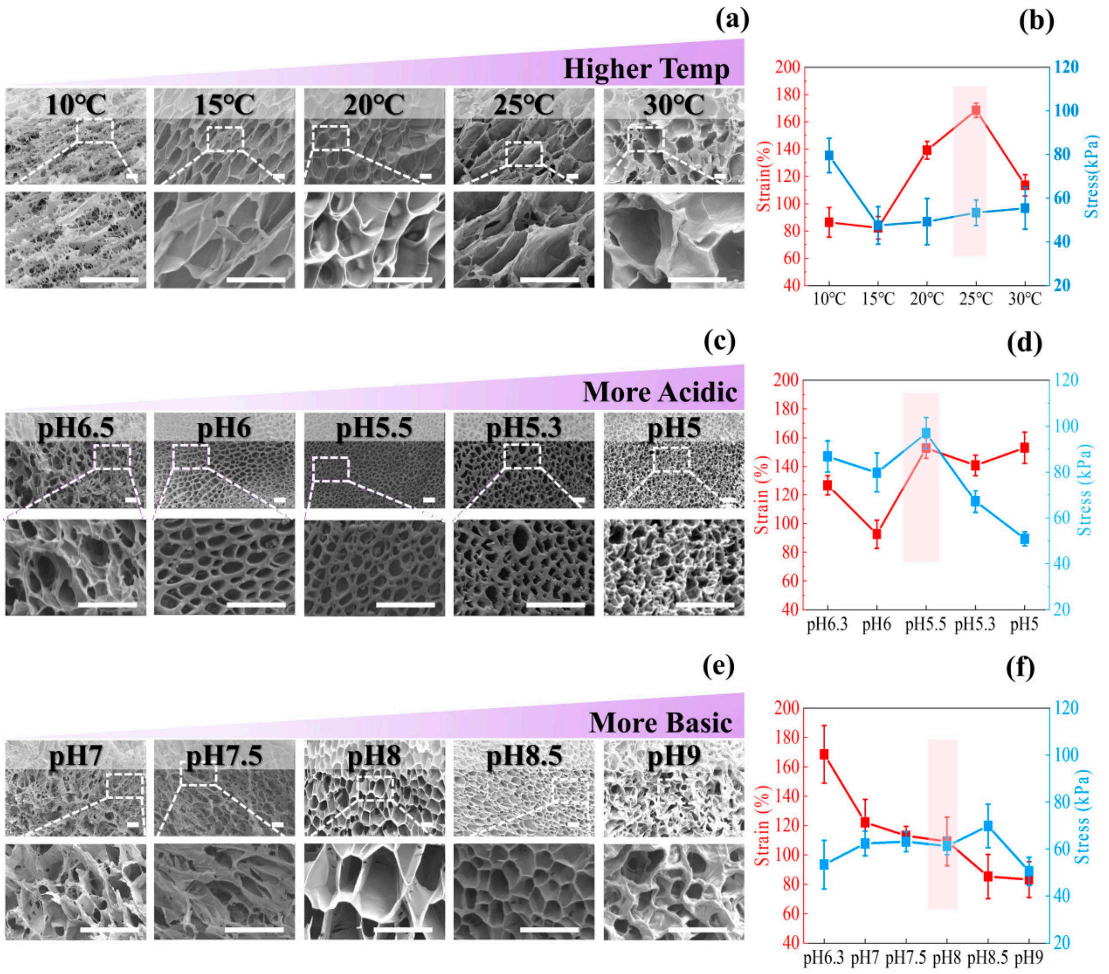

Figure S3. Regulation of SF-Seri/RB hydrogel microstructure and mechanical properties by environmental temperature and precursor pH.

(a, b) Effects of temperature on the hydrogel network morphology and mechanical performance;

(c, d) Changes in hydrogel structure and mechanics under acidic precursor conditions;

(e, f) Structural and mechanical responses of hydrogels under basic precursor conditions.

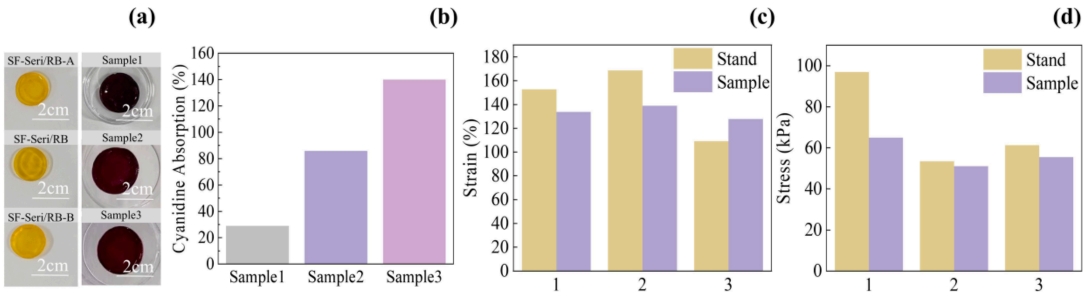

Figure S4. Anthocyanin loading performance and its effect on the mechanical properties of hydrogels.

(a) Photographs of hydrogels after treatment in anthocyanin solution;

(b) Comparison of anthocyanin absorption efficiency;

(c) Strain variation of hydrogels after anthocyanin loading;

(d) Stress variation of hydrogels after anthocyanin loading.

Table S1. Hue values and their corresponding perceptual color categories

Color	Hue Range	Description
Red	0–15, 240–255	Typical red zone; Hue wraparound ends
Orange	16–40	Warm tone between red and yellow
Yellow	41–70	Bright hue representing warm-neutral
Green	71–127	Broad range; highly sensitive to eye
Cyan	128–170	Cool tone between green and blue
Blue	171–210	Cold tone, often for water indicators
Purple	211–239	Transition zone from blue to red
